# Biopolymers Regulate Silver Nanoparticle under Microwave Irradiation for Effective Antibacterial and Antibiofilm Activities

**DOI:** 10.1371/journal.pone.0157612

**Published:** 2016-06-15

**Authors:** Palaniyandi Velusamy, Chia-Hung Su, Govindarajan Venkat Kumar, Shritama Adhikary, Kannaiyan Pandian, Subash C. B. Gopinath, Yeng Chen, Periasamy Anbu

**Affiliations:** 1 Department of Biotechnology, School of Bioengineering, SRM University, Kancheepuram, Tamil Nadu, India; 2 Department of Chemical Engineering, Ming Chi University of Technology, Taishan, Taipei, Taiwan; 3 Department of Inorganic Chemistry, University of Madras, Guindy Campus, Chennai, Tamil Nadu, India; 4 Institute of Nano Electronic Engineering, Universiti Malaysia Perlis, Kangar, Perlis, Malaysia; 5 School of Bioprocess Engineering, Universiti Malaysia Perlis, Arau, Perlis, Malaysia; 6 Department of Oral Biology and Biomedical Sciences, Faculty of Dentistry, University of Malaya, Kuala Lumpur, Malaysia; 7 Department of Biological Engineering, College of Engineering, Inha University, Incheon, Republic of Korea; Institute for Materials Science, GERMANY

## Abstract

In the current study, facile synthesis of carboxymethyl cellulose (CMC) and sodium alginate capped silver nanoparticles (AgNPs) was examined using microwave radiation and aniline as a reducing agent. The biopolymer matrix embedded nanoparticles were synthesized under various experimental conditions using different concentrations of biopolymer (0.5, 1, 1.5, 2%), volumes of reducing agent (50, 100, 150 μL), and duration of heat treatment (30 s to 240 s). The synthesized nanoparticles were analyzed by scanning electron microscopy, UV-Vis spectroscopy, X-ray diffraction, and Fourier transform infrared spectroscopy for identification of AgNPs synthesis, crystal nature, shape, size, and type of capping action. In addition, the significant antibacterial efficacy and antibiofilm activity of biopolymer capped AgNPs were demonstrated against different bacterial strains, *Staphylococcus aureus* MTCC 740 and *Escherichia coli* MTCC 9492. These results confirmed the potential for production of biopolymer capped AgNPs grown under microwave irradiation, which can be used for industrial and biomedical applications.

## Introduction

Nanotechnology is currently a rapidly expanding area of science because of the wide potential applications of nanoparticles in various disciplines [1–6]. Among different soluble and insoluble nanoparticles, metal nanoparticles including silver (Ag), gold (Au), and copper (Cu) play important role in nanotechnological studies and exhibit a wide range of interesting optical and electronic properties in the biological sciences because of their brilliant colors and inherent antibacterial activity [7–9]. In addition, silver nanoparticles (AgNPs) have become very important owing to their unique and remarkable electronic, optical, catalytic, mechanical, heat transfer, and conductivity, and their potential for use in biomedical applications [10–14]. Soluble silver has been reported to have antimicrobial activity and used in treatment of nicotine addiction, mental illness, gastroenteritis, epilepsy, and diseases including gonorrhea and syphilis. The antimicrobial activity of AgNPs makes them useful in cosmetics, textiles, household appliances, electronics, food products, diagnosis, and drug-delivery. Because of the dissolved oxygen AgNPs in aqueous systems are more toxic to the cells than bulk silver [15]. Due to stability and the solubility nature of silver, it has also been used as a replacer in agrochemicals [16].

Several methods have been demonstrated for preparation of silver nanoparticles via chemical reduction, photochemical reduction, reverse micelles processes, electrochemical, reflux sonochemical methods, UV photolysis, microwave dielectric heating reduction, ultrasonic irradiation radiolysis, solvothermolysis, and green synthesis of metal salts [17–19]. Silver nanoparticles prepared using such routes must have good dispersibility and thermal stability, which are important factors for use in industrial applications. Utilization by reducing and protecting molecules or organic capping of molecules during synthesis of nanoparticles is a common approach to synthesis of stable nanoparticles without aggregation problems [20–22]. Currently, green synthesis of silver nanoparticles has opened up a new route for production of different sized and shaped nanoparticles without use of harmful chemical reducing agents. In this regard, plant extracts, natural antioxidants, green solvents, amino acids, and glucose and its derivatives are commonly used as reducing agents [23–25]. The silver nanoparticles produced by use of this technique have been utilized to study antimicrobial activity [26], dye decoloration, and electrochemical degradation of chloro-organic compounds [27]. Microwave synthesis of silver nanoparticles is another green method for their production [28].

Three important factors that must be considered during green synthesis of AgNPs are (i) use of green solvents, (ii) use of an ecological, benign reducing agent, and (iii) use of a safe material as a stabilizer. One green method for preparation of AgNPs is the polysaccharide method, which employs H_2_O as a green solvent and polysaccharides as capping agents. Raveendran et al. [7] reported on the first absolutely green synthesis of AgNPs with H_2_O, starch and D-glucose as the solvent, reducing and capping agent, respectively. Based on modification of this method, production of AgNPs has been demonstrated using different sugars and biopolymers (starch, gelatin) as reducing agents [29, 30].

Sodium alginate (SA), which is derived from brown marine algae, is a naturally occurring poly-anionic polysaccharide consisting of 1, 4-linked β-D-mannuronic and α-L-guluronic residues in different proportions. Carboxymethyl cellulose (CMC) macromolecules are composed of chemically altered chains of cellulose comprising carboxyl and reducing groups. The non-reducing and anionic features support the use of CMC and SA as reducing and stabilizing agents for synthesis of AgNPs. Solubilized CMC and SA negative charges assist in the attraction of positively charged cations of silver to chains of polymer followed by reduction with available reducing groups. Both are economical, biocompatible, and environmentally non-threatening biopolymers with various applications in the biotechnology industry as non-toxic food preservatives, condensing agents, crystallizing agents, and colloidal stabilizers [31, 32]. Alginate has wide applications in wound curative materials, bioactive agent delivery, and cell transplantation due to its structural similarities to extracellular conditions of living tissues [9].

Microwave synthesis is a rapid and time saving green synthesis method for preparation of nanoparticles. In the current study, we used the microwave method to synthesize AgNPs because of the different advantages of this approach and its capacity for rapid synthesis of nanoparticles. The microwave synthesis method also ensures uniform heating of the reaction mixture, and herein it was employed in the aqueous synthesis of AgNPs using various biopolymers as stabilizing agents in the presence of trace amounts of aniline as reducing agents. In addition, the synergistic effects of biopolymer capped AgNPs in the presence of trace amounts of polyaniline as the oxidation product were tested for antibacterial behavior against Gram-negative (*Escherichia coli*) and Gram-positive (*Staphylococcus aureus*). *E*. *coli* and *S*. *aureus* are more common nosocomial pathogens and show resistance to a majority of commercially available antibiotics [33]. The emerging nanomaterial based approach to control of biofilm formation has shown promise as they are less resistant against metal nanoparticles than commercial antibiotics.

## Materials and Methods

### Materials

Silver nitrate (AgNO_3_ ≥99.0%), carboxymethyl cellulose (CMC) sodium salt (Mw ~90,000), sodium alginate (SA), and aniline were obtained from Sigma-Aldrich (USA). Clinical pathogens including *Escherichia coli* MTCC 9492 and *Staphylococcus aureus* MTCC 740 were obtained from Microbial Type Culture Collection (MTCC), Chandigarh, India. Muller-Hinton broth and agar were purchased from Himedia, India. Deionized Milli-Q H_2_O was used during the course of the experiments.

### Microwave synthesis of biopolymer capped AgNPs

The experiment was performed under different experimental conditions, with varying concentrations of biopolymer (0.5, 1, 1.5 and 2%), volumes of reducing agent (aniline) (50, 100, 150 μL) and times of heat treatment (30 s to 240 s). In a typical experiment, 32 mL of water, 1 mL of 0.04 M AgNO_3_ and 1.6 mL of CMC/SA at different concentrations and different volumes of aniline were mixed at room temperature and heated in a microwave at 80% power for different time intervals. The samples were then cooled to room temperature. Following optimization, the samples were characterized.

### Characterization

Samples were analyzed by scanning electron microscopy (SEM) (Quanta FEI 200, USA) to examine the morphological and structural features of the capped AgNPs at 20.00 kV. UV-visible spectra of coated nano-silver hydrogel were measured in the range of 200 to 1000 nm using an UV-visible spectrophotometry (Shimadzu, Japan). X-ray diffraction analysis was performed using an X’PertPro A Analytical X-ray diffractometer with Cu Kα radiation (k = 1.54056 Å) in the range of 30 to 80 (2 θ values) at 40 keV and compared with the JCPDS. Fourier transform infrared spectroscopy (FTIR) from Perkin-Elmer (USA) was performed for analysis of the functional groups on the coated AgNPs in the range of 400 to 4000 cm^-1^ at a resolution of 4 cm-^1^.

### Antibacterial assay

Antibacterial activity of the capped AgNPs was tested against Gram-positive bacteria (*S*. *aureus*) and Gram-negative bacteria (*E*. *coli*) using the disc agar diffusion method [34]. The bacterial suspensions (10^6^ CFU/ml) were swabbed on Mueller-Hinton agar media plates using sterile cotton buds, followed by placement of 6 mm sterile discs on the plates. Biopolymer capped AgNPs suspensions were then loaded into the discs at different volumes (6, 9, 12, 15 μL) with incubation of the plates at 37°C for 24 h. Following incubation, the zone of inhibition was measured using the HI-antibiotic zone scale^TM^.

The effect of different concentrations of CMC@AgNPs and SA@AgNPs on biofilm formation was tested. LB broth (100 mL) was inoculated using a single bacterial colony and incubated overnight at 37°C. The culture was then diluted with freshly autoclaved LB broth to a concentration of 10^6^ CFU/mL, and 2 mL bacterial suspensions were pipetted into polystyrene 24 well plates in the presence of different concentrations (8, 16, 32, 64, and 128 μg/mL) of CMC@AgNPs and (SA@AgNPs. Chloramphenicol (20 μg/mL) was used as a known antibiotic and double sterilized millipore water used as a control. Bacteria were allowed to grow aerobically for 48 h at 37°C, followed by removal of the planktonic culture and the wells were washed thoroughly with 1X PBS (phosphate-buffer saline) to discard non-adherent bacterial cells; 1 mL of crystal violet dye (0.1%) was added and mixed in each well with incubation at room temperature for 15–20 min for staining of the adhered biofilm. The dye was then discarded and the wells were washed thoroughly two times with millipore water for removal of excess unbound dye, followed by addition of 1 mL of 33% acetic acid to solubilize the dye bound to the biofilm. The intensity of the stained suspension was measured at 575 nm absorbance using 33% glacial acetic acid as a control.

## Results and Discussion

Biopolymers have many applications including catalysis [11], antibacterial studies, and sensors [14, 35], and they are non-toxic, inexpensive, and biocompatible [36, 37]. This enhanced biocompatibility of synthesized enables their use in various biomedical applications [3]. Compared to other chemical and physical methods for synthesis of AgNPs, microwave assisted synthesis of biopolymer capped AgNPs is an easy and effective method for increasing their stability and for manipulation of particle size and growth rate [38, 39]. The advantage of microwave-mediated synthesis over other physical treatment is rapid initial heating which improves the kinetics of the reaction by one or two orders of magnitude, homogenous heating, and higher yields. In addition, the microwave synthesis approach is simple, fast, and environment-friendly. Hence, the method described herein is considered a green chemistry approach for synthesis of AgNPs for large scale production. A summary of the comparison of microwave assisted methods with other physical methods and their morphological size is shown in Table 1. The major objective of this work was to synthesize stable AgNPs and determine their antibacterial behavior and synergistic properties. Two different biopolymer capped AgNPs were synthesized under different experimental conditions, and we examined the inherent antibacterial activity of biopolymer capped AgNPs. The synergistic effects observed in this study could lead to eradication of some antibiotic resistant bacteria.

**Table 1:** Comparison of the microwave assisted methods with other physical methods and summary of their morphological size.

Method of synthesis AgNPs	Size of the AgNPs	Morphology of the AgNPs	References
**Microwave annealing**	< 50 nm	Spherical shape	[40]
**Thermal annealing**	5–45 nm; 7–20 nm	Spherical shape	[41, 42]
**Ion irradiation**	5–25 nm; <10 nm	Almost spherical shape	[43,44]
**UV irradiation**	<50 nm	Spherical or ellipsoidal or triangular	[45]
**γ-irradiation**	<20nm; <30nm	Mostly spherical <10nm	[46,47]

### Microwave synthesis of biopolymer capped AgNPs

A stable dispersion of biopolymer capped AgNPs in aqueous medium was observed using the microwave irradiation method. A sudden color change was observed within a few minutes of heating, indicating the formation of biopolymer capped AgNPs. A yellow colored solution was obtained when SA was used as a stabilizing agent, which became a dark brownish orange upon cooling. For CMC@AgNPs, the solution turned yellow after standing at room temperature. Synthesis was repeated under various experimental conditions to optimize the experimental procedures and observe the formation of characteristic AgNPs. This biopolymer capped AgNPs were isolated by concentration using lyophilization. Intrinsic properties of AgNPs generated with polymer are influenced by the size, shape, and structure as reported by Bonnemann and Richards [40]. The interplay mechanism is mainly due to the morphologies forming cluster nucleation, its growth and assembly during synthesis [41]. Theoretically the repulsion energy among nanoparticles decreases with the concomitant increment in ionic strength. Surface and bulk quantity and surface atoms are the predominant considerations for the size influences on the thermodynamic properties of silver or other nanoparticles. The dried, powdered nanoparticles were studied by FTIR, XRD, and SEM analysis.

### Characterization

**Fig 1:**
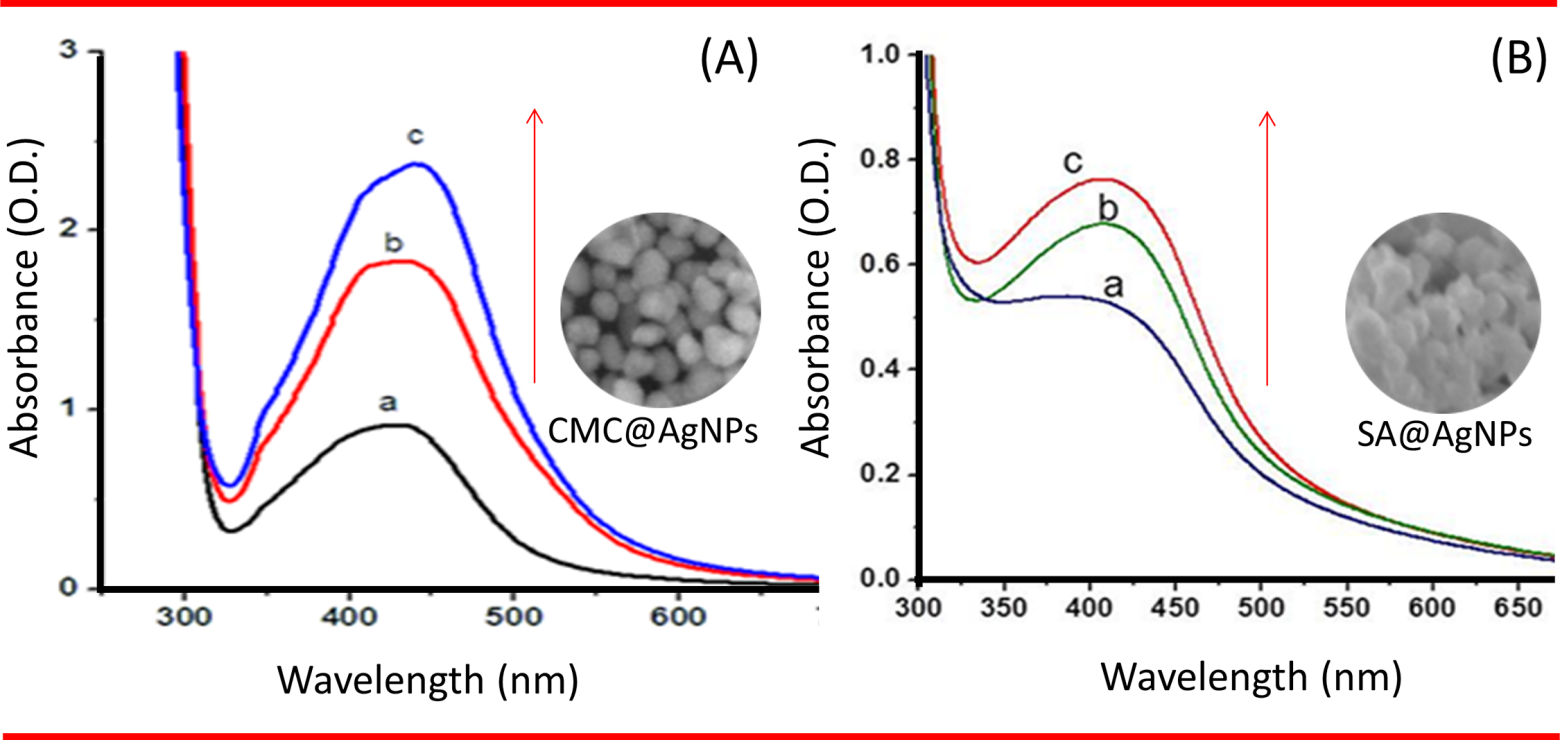
Ultraviolet–Visible spectroscopy. (A) CMC@AgNPs at (a) 50 μl, (b) 100 μl, (c) 150 μl and (B) SA@AgNPs at 50 μl (a), 100 μl (b) and 150 μl (c) of aniline. Arrow indicates direction of spectral changes.

In CMC@AgNPs, characteristic peaks were observed at 440, 430, and 425 nm for different volumes of aniline (150, 100, and 50 μL, respectively) as shown in Fig 1A. The absorption bands may be represented by the surface plasmon resonance (SPR) of AgNPs. The broad nature of the peaks indicated the formation of different shaped AgNPs, which was further confirmed by the SEM images. Based on the results, 100 μL of aniline was considered the optimal volume. Evaluation of SA@AgNPs showed absorbance peaks at 430, 425, and 415 nm for 150, 100, and 50 μL of aniline, respectively (Fig 1B). The characteristic bands confirm the synthesis of silver crystals. A gradual increase in the peaks was observed, inferring an increase in the size of the crystals. Based on these findings, 150 μL of aniline was considered the optimal volume for SA@AgNPs.

**Fig 2:**
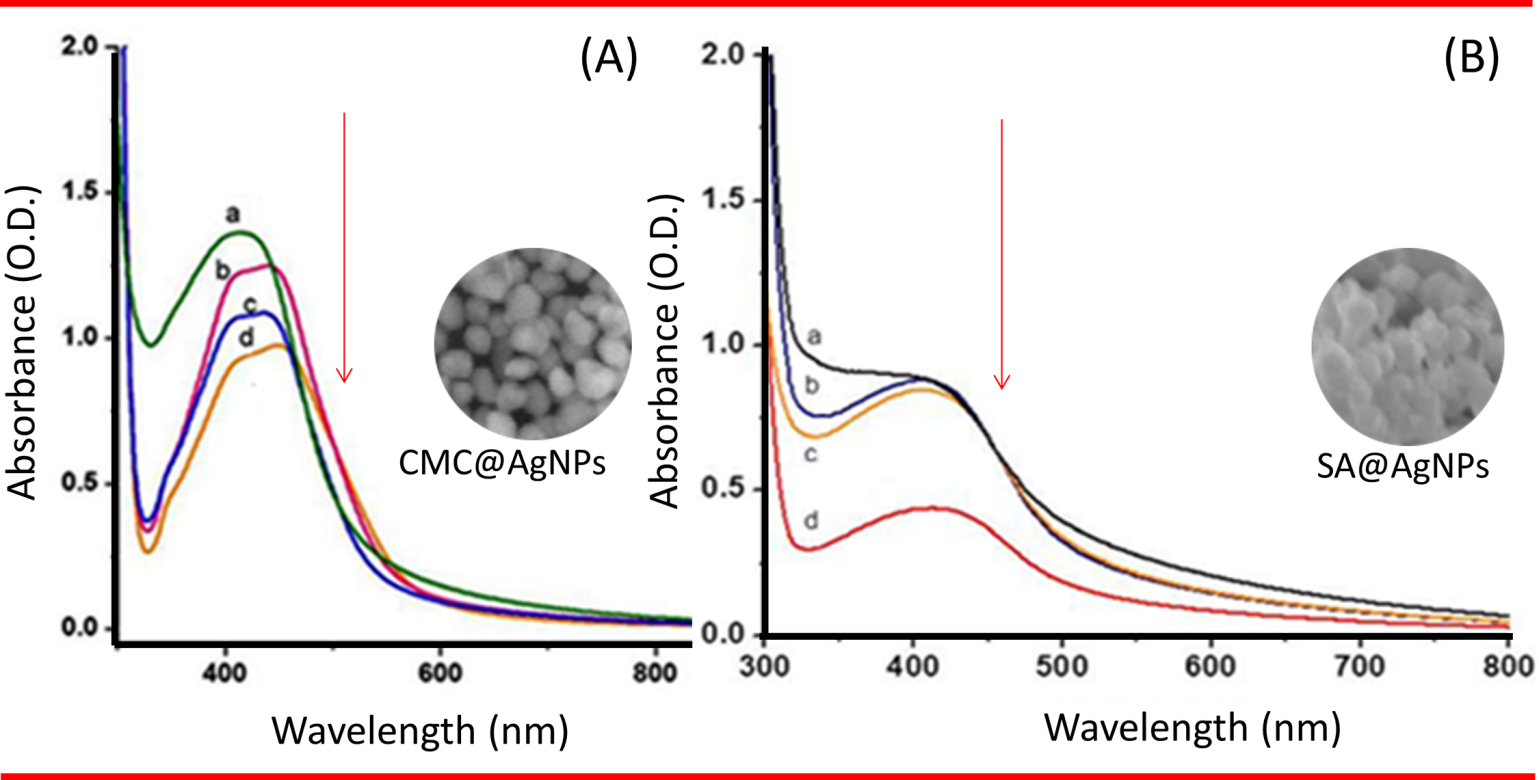
Ultraviolet–Visible spectroscopy. (A) CMC@AgNPs at (a) 0.5%, (b) 1%, (c) 1.5%, and (d) 2% (B) CMC. (II) SA@AgNPs at (a) 0.5%, (b) 1%, (c) 1.5%, and (d) 2% SA. Arrow indicates direction of spectral changes.

**Fig 3:**
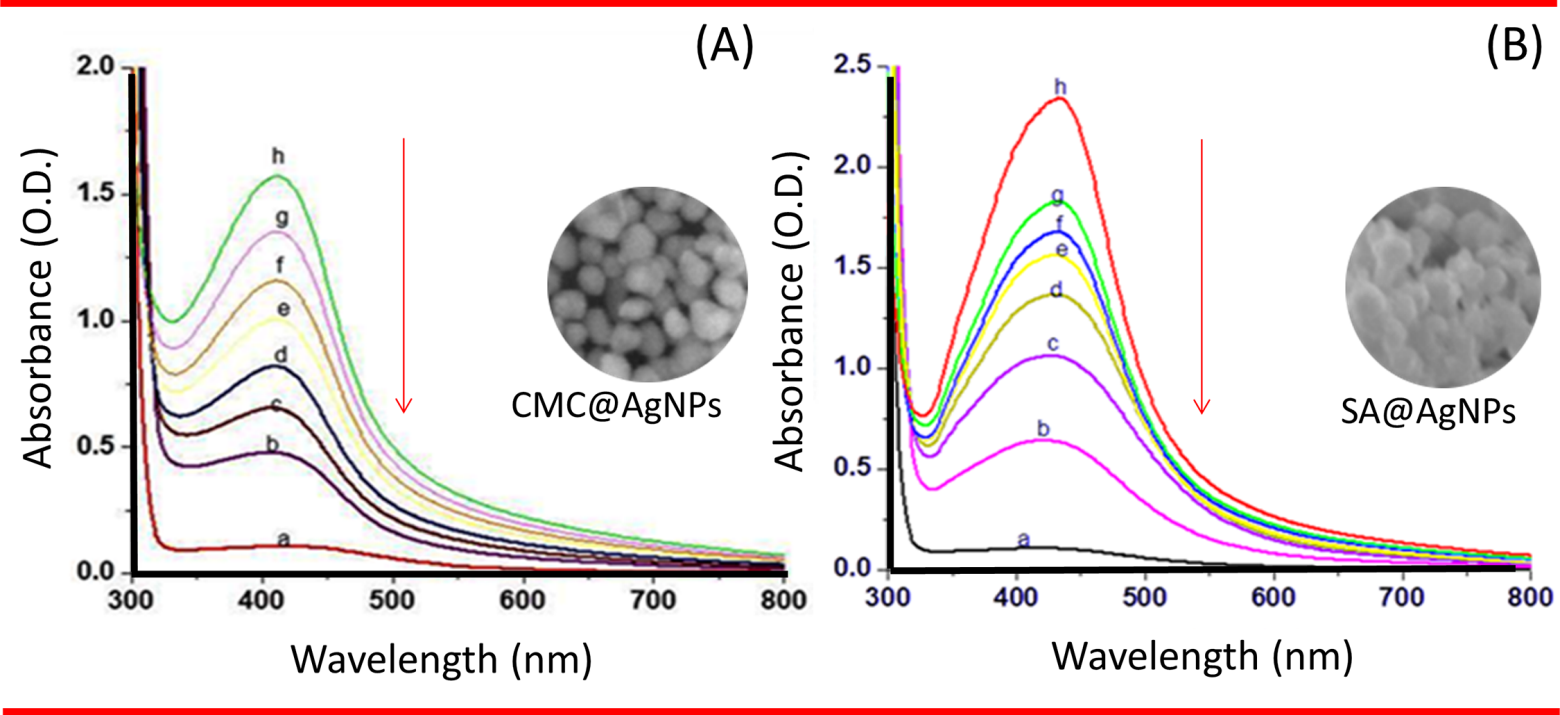
Ultraviolet–Visible spectroscopy. (A) CMC@AgNPs at (a) 30s, (b) 60s, (c) 90s, (d) 120s, (e) 150s, (f) 180s, (g) 210s and (h) 240s. (B) SA@AgNPs at (a) 30s, (b) 60s, (c) 90s, (d) 120s, (e) 150s, (f) 180s, (g) 210s and (h) 240s. Arrow indicates direction of spectral changes.

Characteristic peaks at 415 nm indicating surface plasma resonance of AgNPs were obtained for all concentrations of CMC. Peaks were observed at 415, 435, 440, and 445 nm for 0.5, 1, 1.5, and 2% CMC, respectively (Fig 2A). These peaks signified the SPR of the AgNPs. There was a gradual decrease in absorbance, which implied an increase in size and decrease in the rate of synthesis of the biopolymer capped AgNPs. Hence, 1% (w/v) was the minimum percentage of CMC required to coat AgNPs. As shown in Fig 2B, the maximum absorbance was obtained for both 0.5% and 1% SA. Based on these findings, 1% (w/v) SA was considered the optimum percentage for coating AgNPs. The broad nature of the peaks suggested different shaped nanoparticles, which was validated by the SEM images. The UV-Vis spectral data of CMC@AgNPs and SA@AgNPs (Fig 3A & 3B) for different heating times (30 s to 240 s) showed that the absorbance also increased with increasing time of heat treatment. These findings implied that the quantity of nanoparticles synthesized increased with the increase in heating time. Based on these results, heating for 240 s was considered optimal for synthesis of biopolymer capped AgNPs.

**Fig 4:**
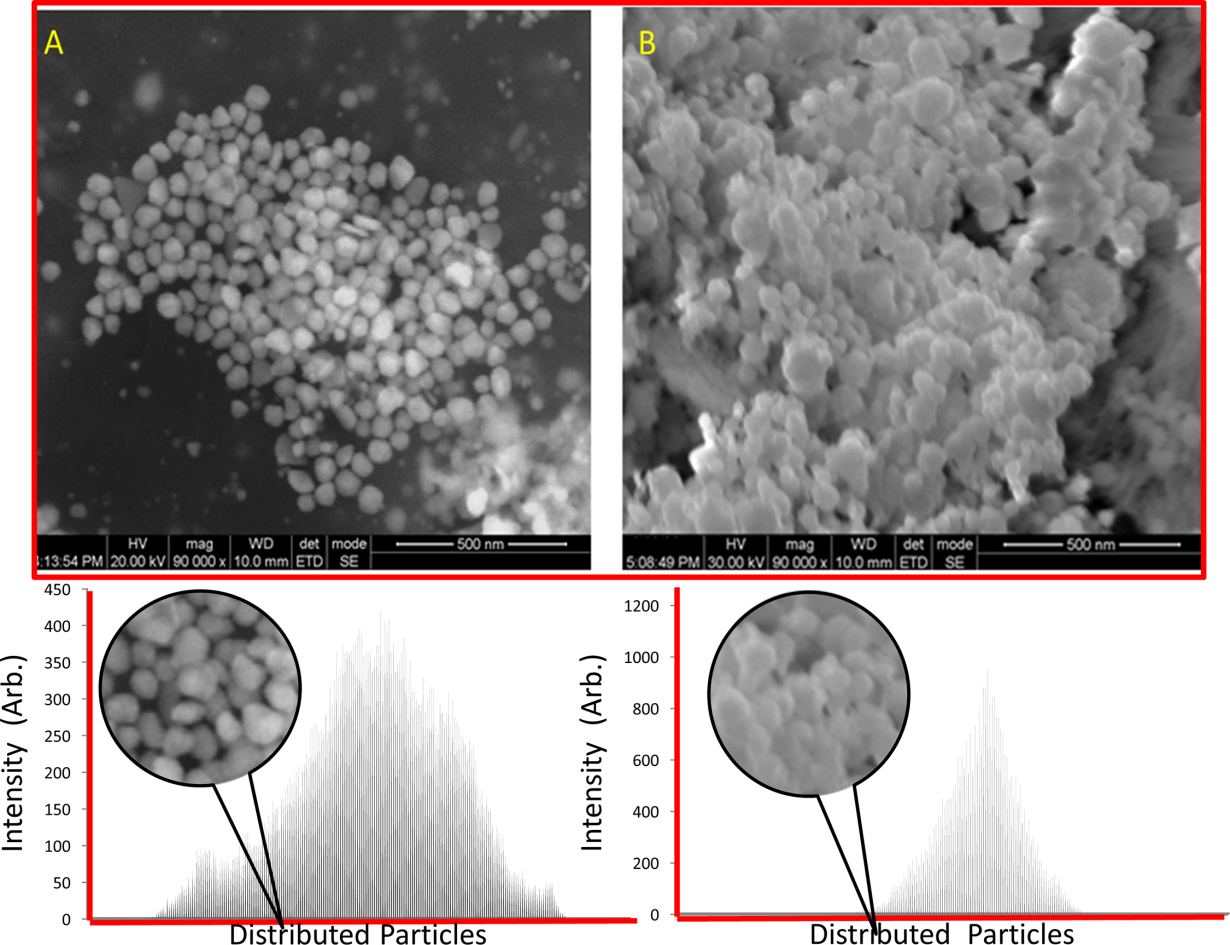
Scanning electron microscopic images. (A) CMC@AgNPs and (B) SA@AgNPs.

Following optimization, the biopolymer capped AgNPs were synthesized and characterized by SEM for morphological analysis. The SEM images indicated that the polygon shaped CMC@AgNPs had a size of 50–100 nm. For SA@AgNPs, nanoparticles with encapsulation of SA were observed (Fig 4A & 4B) in the range of 100–150 nm. No aggregation of AgNPs was observed in both cases. The distribution patterns were analyzed by scanning using ImageJ software, which showed a spacious distribution for CMC@AgNPs, whereas close distribution was observed for SA@AgNPs (Lower panels in Fig 4). Synthesis of AgNPs usually involves a two-stage process, formation of an atom and atom polymerization. Initially, biopolymers reduce the percentage of metal ions in the solution and catalyze with the reduction of remaining metal ions, where the atoms formed act as a nucleation core. Consequently, the atoms amalgamate, leading to metal cluster formation, with formation of larger particles as the surface ions that are continuously reduced until high standards of nucleation are attained.

**Fig 5:**
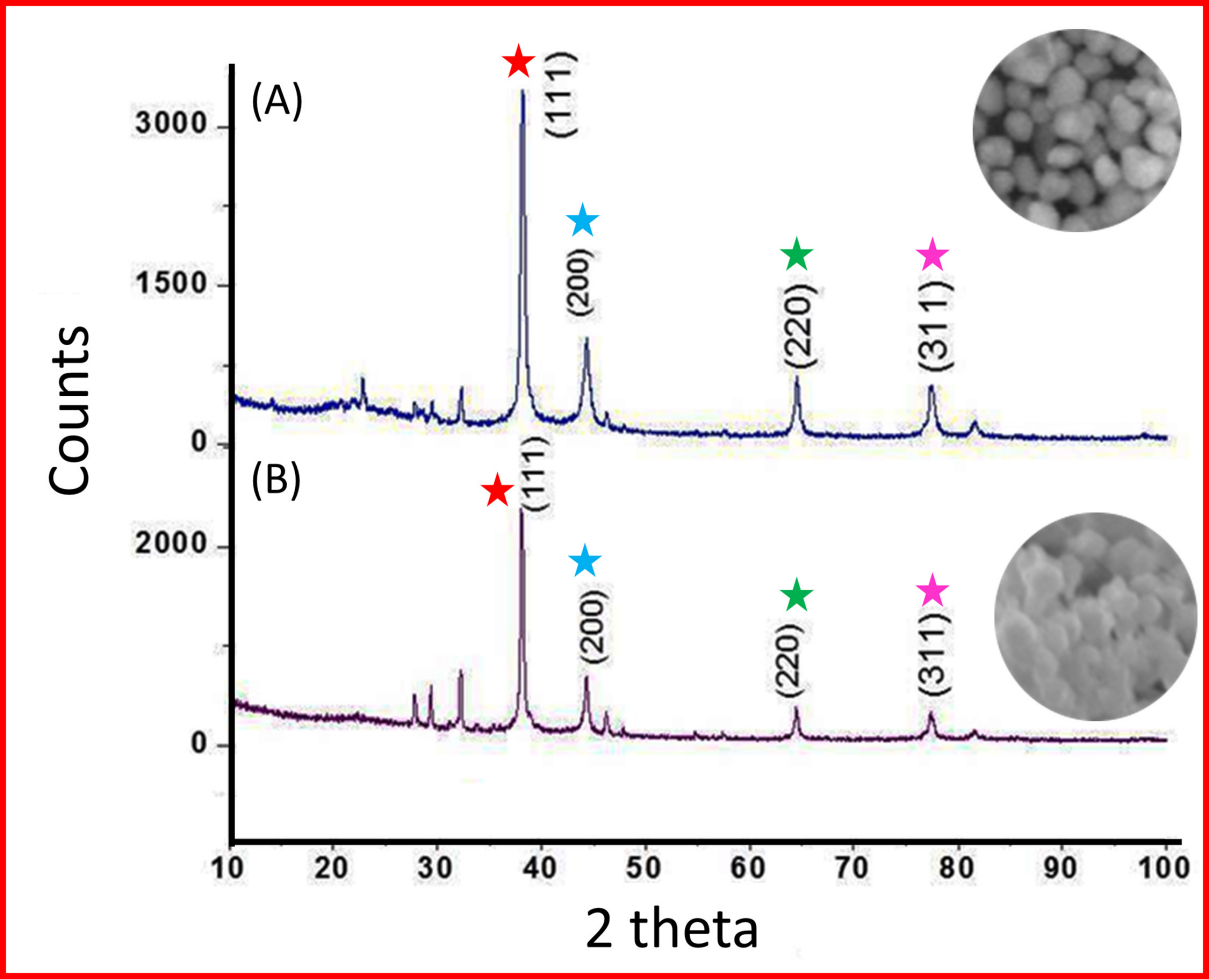
X-ray diffraction analysis. (A) CMC@AgNPs and (B) SA@AgNPs. Peak comparison is indicated by respective colored stars.

In addition, the polymer interacts with the metal particles, which stabilizes and prevents additional coalescence [42–50]. The results of XRD analysis data for CMC@AgNPs and SA@AgNPs are shown in Fig 5. Distinct characteristic peaks at 38.1°, 44.3°, 64.5°, and 77.4° were obtained representing the crystallographic planes (111), (200), (220), and (311), respectively, of the silver nanocrystals [43, 46]. The lattice constant calculated from the diffracted spectrum was *a =* 4.0857 Å and the resultant data matched that in JCPDS file no. 01-087-0717. The mean crystallite diameter (D) of the biopolymer capped AgNPs formed in the reduction process is determined using Scherrer’s equation D_p_ = Kλ/β_1/2_cos θ and is estimated as 87.82 nm and 117.09 nm for CMC@AgNPs and SA@AgNPs, respectively, in which K is the shape dependent Scherrer’s constant (0.94), λ is X-ray wavelength (1.5406 Å), β_1/2_ is X-ray line full width at half maximum, and θ is the Bragg angle. It remains the same with altering experimental conditions. As previously reported by Campi et al. [41] spatial correlations between primary particles and the dynamic fractal geometry with time evolution influenced the polymer-silver matrix and determined the mechanism of aggregation and the morphological features of the nanostructures formed. This might be the reason for the intense diffraction line of Ag with polymer aggregation.

**Fig 6:**
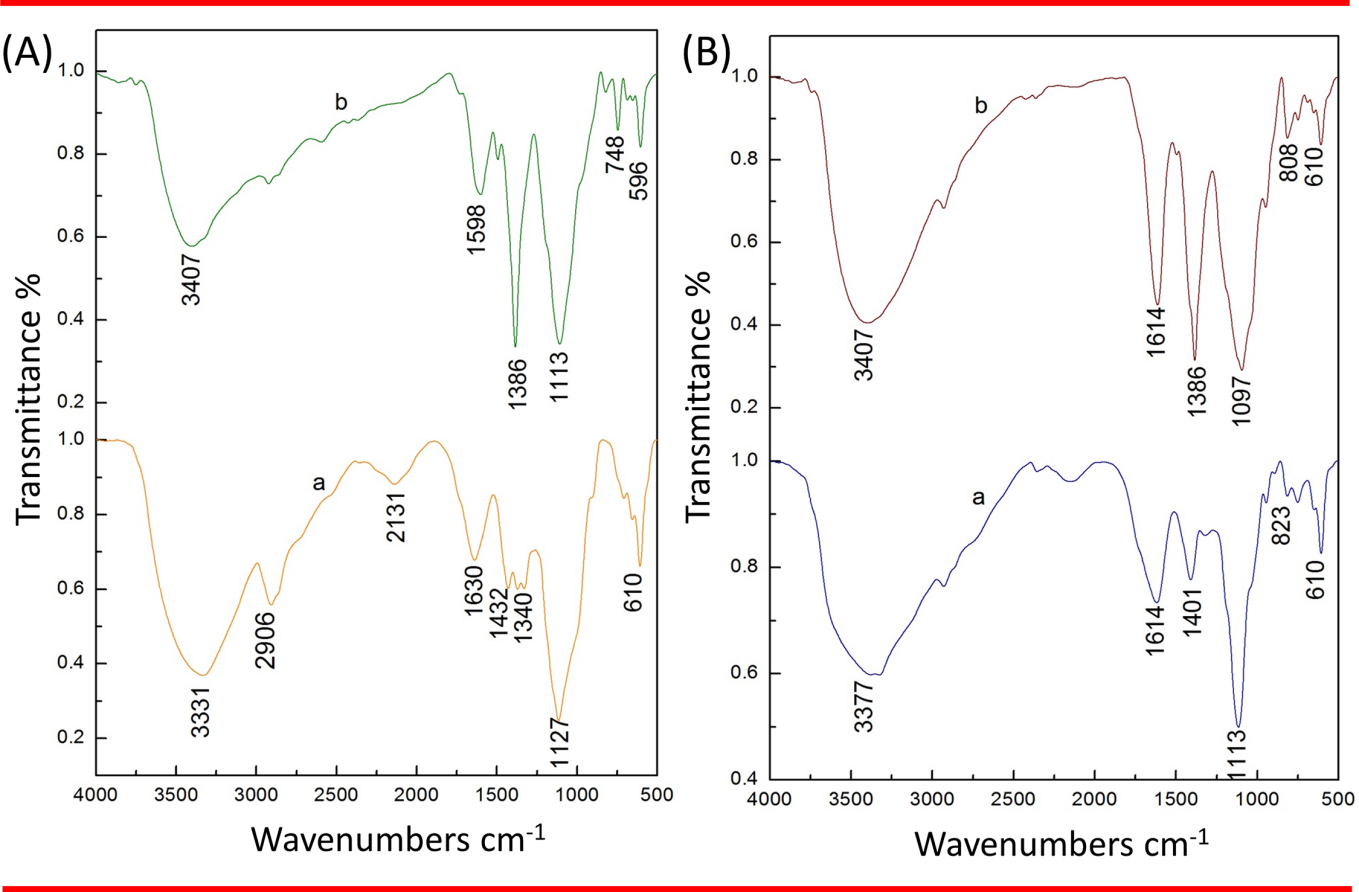
Fourier transform infrared spectra. (A) CMC—(a) Pure CMC and (b) CMC@AgNPs. (B) SA—(a) Pure SA and (b) SA@AgNPs.

The peak at 1432 cm^-1^ was absent from CMC@AgNPs, indicating involvement of symmetric COO^−^in formation of the coating. The peak shift to 1598 cm^-1^ from 1630 cm^-1^ was due to involvement of C = C in CMC hydrogel formation on the surface of the AgNPs (Fig 6A). In SA@AgNPs, the FTIR spectra (Fig 6B) showed peaks at 3377 cm^-1^and 3407 cm^-1^ corresponding to OH groups, as well as a peak at 1614 cm^-1^ corresponding to COO−groups. A blue shift was observed from 1401 cm^-1^ to 1386 cm^-1^, which confirmed participation of the CO group in creation of the coating on the surface of the AgNPs. A peak shift from 1113 cm^-1^ to 1097 cm^-1^ confirmed involvement of the CO group in formation of the biopolymer network. These findings are in agreement with those of previous studies [8, 37].

### Antibacterial assay of biopolymer capped AgNPs

The bacterial cell membrane consists of sulfur-containing proteins and phosphorus containing nuclear elements, which are the target sites for AgNPs as Ag has a higher tendency to bind with these elements [51, 52]. AgNPs form a region of low molecular weight in the core of the bacterial cell and attack the respiratory chain process, resulting in cell death. AgNPs further release silver ions from bacterial cells, increasing bactericidal activity [53–56].

Biopolymer capped AgNPs exerted effective antibacterial activity against both Gram-positive bacteria (*S*. *aureus*) and Gram-negative bacteria (*E*. *coli*). The inhibition zones (mm) around each well for different concentrations of AgNPs are shown in Table 2. Comparison of the maximum inhibitory zones for both Gram-negative and Gram-positive bacteria for different biopolymer capped AgNPs against gentamicin (15 μg/mL) found that CMC@AgNPs and SA@AgNPs were effective in inhibiting growth of Gram-negative and Gram-positive bacteria. CMC@AgNPs showed more activity toward Gram-negative than Gram-positive bacteria. Several mechanisms of action by silver ions were thought to include structural changes in the cell wall of bacteria and interactions with thiol groups in proteins and enzymes and interruption of DNA replication due to damage of the DNA [22].

**Table 2:** Antibacterial activities of different concentrations of CMC@AgNPs and SA@AgNPs by disc agar diffusion method.

Bacteria	Concentration of CMC@AgNPs and SA@AgNPs (μg/mL)	Inhibition zone (mm) Mean of three replicates CMC@AgNPs	Inhibition zone (mm) Mean of three replicates SA@AgNPs
*E*. *coli*	6 9 12 15	11 11.5 12 12.5	8 8.5 10 10.5
*S*. *aureus*	6 9 12 15	7.5 9 12 12.5	8.5 9.5 12.5 13.5

**Fig 7:**
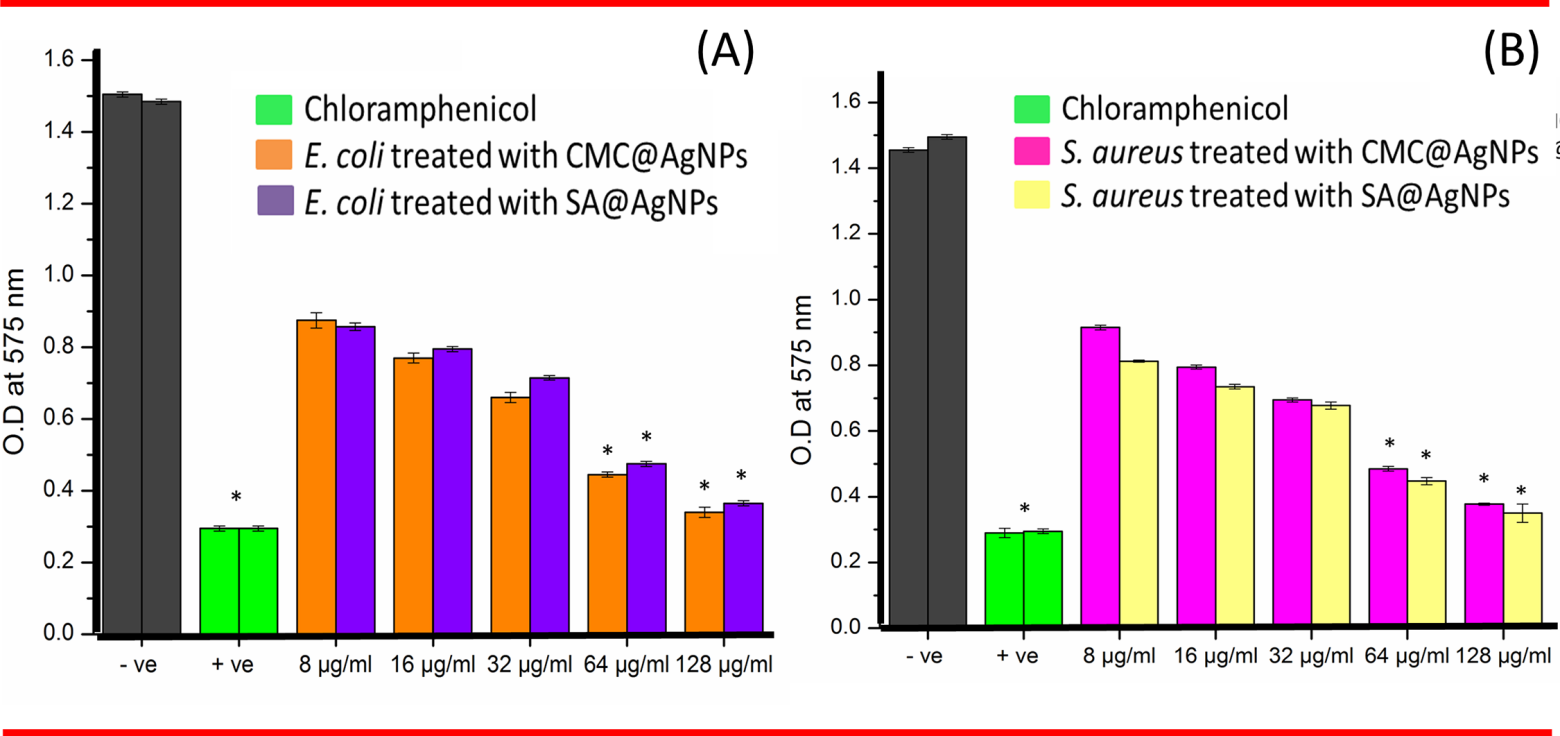
**(A) Antibiofilm efficacy of** CMC@AgNPs **and (B)** SA@AgNPs **against both *E*. *coli* and *S*. *aureus*.** Error bars represent the standard deviation. * Significantly different (P<0.05) from the positive control.

Similarly, in the biofilm assay CMC@AgNPs was more active against *E*. *coli* and SA@AgNPs were more active against *S*. *aureus* (Fig 7A & 7B). Importantly, the increase in the concentration of CMC@AgNPs and SA@AgNPs caused a significant (P <0.05) increase in biofilm inhibition relative to that grown in the negative control. The higher resistance observed in Gram-negative bacteria compared with Gram-positive bacteria was due to differences in cell membrane polarity. Gram-positive bacteria have a thicker peptidoglycan layer consisting of chains of short peptide cross linked linear polysaccharide, which restricts the entry of CMC@AgNPs into cells, preventing cell damage. However, for Gram-negative bacteria, CMC@AgNPs attach easily to the thinner peptidoglycan layer causing rupture of the cell wall, resulting in cell death. SA@AgNPs showed more activity toward Gram-positive than Gram-negative bacteria, which might have been due to the cell membrane polarity response to SA@AgNPs. Shilpa Sharma et al. [57] reported that Gram-negative bacteria have a higher negative surface charge compared with Gram-positive bacteria, in turn the alginate AgNPs will interact less with Gram-negative bacteria compared with Gram-positive bacteria. CMC and SA reportedly have no antibacterial inhibition activity [58, 59], however they direct AgNPs to attack bacteria. Several studies elucidating the antibacterial effect have been proposed. Different antibacterial mechanisms of action include interference with cell wall synthesis, inhibition of protein synthesis, interference with nucleic acid synthesis, and inhibition of a metabolic pathway [60–63].

## Conclusion

The microwave method is a green approach to synthesis of different biopolymer capped AgNPs in a specific minute, instead of hours or even days. The microwave method is currently considering a rapid increase in acceptance as a technique for enhancing facile synthesis of AgNPs using CMC and SA. Formation of polymer capped AgNPs under different experimental conditions was observed by UV-Vis spectroscopy and optimized. XRD analysis confirmed the purity of the samples. Successful capping of the AgNPs with the biopolymers was confirmed by comparing the FTIR spectra of pure biopolymers to those of biopolymer capped AgNPs. The size of the synthesized CMC@AgNPs and SA@AgNPs was approximately 100–150 nm. As shown in our results, polymer capping on the silver showed transition changes due to alterations in absorbance, and, upon changing the conditions, clear transition changes were observed between alginate and cellulose capping on AgNPs. Different biopolymer capped AgNPs showed potent antibacterial activity against both Gram-negative and Gram-positive bacteria. However, CMC@AgNPs were more potent at inhibiting the growth of Gram-negative bacteria than Gram-positive bacteria, and SA@AgNPs showed more inhibition toward Gram-positive than Gram-negative bacteria. These biopolymers would protect humans from the toxicity of AgNPs if used for biomedical applications. In the case of industrial applications, biopolymer capped AgNPs can be used as surface coatings to prevent bacterial adhesion, especially in food packing applications. In addition, the heating methods can address the problems of heating in homogeneity in conventional thermal techniques, as it provides increased reaction kinetics, rapid initial heating, clean reaction products with rapid consumption of starting materials, and higher yields. Hence, biopolymer capped AgNPs can be used for industrial food and biomedical applications.
